# Atomic Layer Deposition for Perovskite Solar Cells: Interface Engineering, Stability Enhancement, and Future Prospects

**DOI:** 10.3390/nano15211674

**Published:** 2025-11-04

**Authors:** Xuanya Liao, Youquan Jiang, Lirong Wang, Jiulong Li, Zhuoran Hou, Kwang Leong Choy, Zhaodong Li

**Affiliations:** 1 School of Integrated Circuits, Wuhan University, Wuhan 430072, Chinayouquan_jiang@whu.edu.cn (Y.J.); jiulongli@whu.edu.cn (J.L.); 2 Suzhou Key Laboratory of Advanced Sustainable Materials and Technologies, The Environmental Research Center, Division of Natural and Applied Sciences, Duke Kunshan University, Kunshan 215316, China; 3 The Institute of Technological Sciences, Wuhan University, Wuhan 430072, China; 4 School of Power and Mechanical Engineering, Wuhan University, Wuhan 430072, China

**Keywords:** perovskite solar cells, atomic layer deposition, stability, interface engineering

## Abstract

Perovskite solar cells (PSCs) have achieved rapid progress in recent years owing to their high-power conversion efficiency (PCE), low cost, and processability. However, poor device stability and carrier recombination remain significant obstacles to further development. Atomic layer deposition (ALD), with its atomic-level control over film thickness, excellent uniformity, and interfacial engineering capability, has attracted considerable attention in PSC research. This review summarizes the applications of ALD in PSCs, including low-temperature synthesis (typically below 350 °C), thickness and composition control (approximately 1 nm per 10 ALD cycles), defect passivation, encapsulation (water vapor transmission rates as low as 10^−6^ g·m^−2^·day^−1^ under optimized conditions), and tandem devices. In addition, the mechanisms by which ALD enhances device efficiency and stability are discussed in depth, and the challenges and future prospects of this technique are analyzed.

## 1. Introduction

Against the backdrop of worsening global climate change and growing energy scarcity, solar cells have received widespread attention as a clean energy solution for power generation. Among them, perovskite solar cells (PSCs) are regarded as one of the most promising next-generation photovoltaic devices due to their outstanding properties [1,2]. Compared with conventional silicon-based solar cells, PSCs not only offer lower fabrication costs and simpler processing but also demonstrate good mechanical flexibility [3,4,5,6]. Since 2009, when organic–inorganic lead halide perovskites were first introduced as sensitizers in dye-sensitized solar cells [7], their PCE has achieved remarkable breakthroughs [8,9]. However, perovskite materials are highly sensitive to external factors such as humidity, heat, and illumination, which can cause degradation [10] and lead to performance losses. Moreover, PSCs still face multiple challenges in terms of long-term operational stability, large-scale fabrication, and further efficiency improvement [11,12,13].

To overcome these challenges, researchers have continuously explored approaches from structural design, process optimization, and defect suppression to interfacial engineering, with the aim of improving overall device performance [14,15,16,17,18,19,20]. Within the PSC architecture, the interface between the perovskite absorber and the electron/hole transport layers plays a critical role in regulating charge separation, transport, and recombination [21,22]. If the interface suffers from energy-level mismatches, structural defects, or impurity-induced recombination pathways, device efficiency will be significantly limited [23,24,25]. Therefore, precise interfacial engineering to enhance carrier extraction efficiency and interface stability is of great importance for improving PSC performance and stability. Atomic layer deposition (ALD), a pulsed chemical vapor deposition technique based on self-limiting reactions, enables uniform, high quality and conformal thin film growth with atomic-scale thickness and composition control over large area at relatively low processing temperatures [26,27,28,29]. This method also allows deposition of dense, pinhole-free, high-quality films at relatively low temperatures [30,31,32,33]. ALD and its high-throughput variants, S-ALD and AP-SALD, are pivotal for depositing ultrathin, conformal, and pinhole-free films across a multitude of advanced applications. Conventional ALD is indispensable in semiconductor fabrication for creating high-κ gate oxides, diffusion barriers, and precise etch stops at the nanoscale [34,35,36,37,38,39,40,41,42]. It is also critical in energy storage, enhancing the performance of lithium-ion battery electrodes and solid-state electrolytes through tailored surface modifications [43,44,45,46]. S-ALD and AP-SALD extend these capabilities to areas requiring rapid, large-area, and cost-effective coating processes [47,48,49,50]. This makes them particularly suited for manufacturing next-generation thin-film photovoltaics, flexible OLED displays, and catalytic coatings [51,52,53,54,55,56,57]. Furthermore, their compatibility with roll-to-roll (R2R) processing opens avenues for the industrial-scale production of barrier layers on flexible packaging and polymers, marking a significant advancement for the scalable integration of functional nanomaterials [43,58,59,60,61,62,63,64].

The aforementioned advantages of ALD also make it particularly attractive for use in high-performance devices such as solar cells. Herein, we review the applications of ALD, S-ALD and AP-SALD for perovskite solar cells, including their potential in low-temperature processing, thickness and composition control, defect passivation, encapsulation, and tandem architectures. Furthermore, we analyze the challenges of integrating ALD into PSC manufacturing and discuss future development directions.

## 2. Device Structures and Challenges of PSCs

### 2.1. Device Structures of PSCs

**Figure 1:**
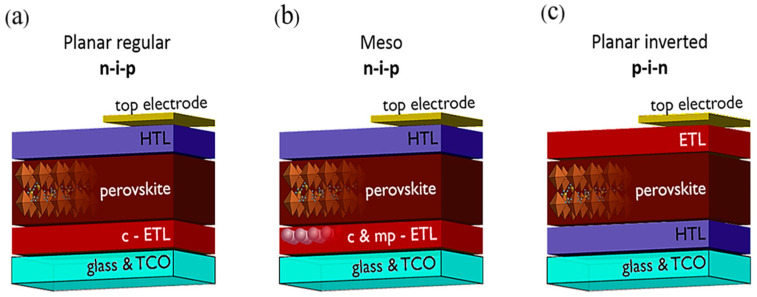
Three PSC architectures, comprising (**a**) planar regular (n–i–p), (**b**) mesoporous (n–i–p), and (**c**) planar inverted (p–i–n) architectures. Reprinted with permission from Ref. [78] Copyright 2018 American Chemical Society.

Depending on the arrangement of the charge transport layers, PSCs can generally be divided into three typical structures: planar regular, mesoporous and planar inverted [20,65]. As shown in Figure 1, various tandem architectures have also been developed to surpass the theoretical efficiency limit of single-junction devices and enhance overall PCE [66,67].

The planar n–i–p architecture is one of the most common PSC designs, where n denotes the electron transport layer (ETL), i the intrinsic perovskite absorber, and p the hole transport layer (HTL). A typical n–i–p structure is FTO/compact ETL/perovskite absorber/HTL/metal electrode [68,69,70]. Compact TiO_2_ or ZnO thin films are commonly used as ETLs [26,71,72], which also act as hole-blocking layers. In contrast, the inverted p–i–n structure positions the HTL directly on the transparent conductive electrode. A representative configuration is ITO/HTL/perovskite/ETL/metal electrode [73,74]. The p–i–n structure offers advantages such as compatibility with low-temperature processing, better operational stability, and reduced hysteresis [75]. The mesoporous architecture typically employs a mesoporous TiO_2_ scaffold, with a configuration of FTO/compact TiO_2_/mesoporous TiO_2_/perovskite/HTL/metal electrode [76,77]. The mesoporous TiO_2_ not only provides electron transport pathways but also improves perovskite crystallinity and interfacial contact [76,78]. Since the open-circuit voltage (V_OC_) of single-junction PSCs is limited by the bandgap, their PCE has a theoretical ceiling. To overcome this limitation, tandem structures have attracted growing interest [79,80,81]. By stacking solar cells with different bandgaps, tandem architectures enable more comprehensive utilization of the solar spectrum, achieving an optimal balance between V_OC_ and short-circuit current density (J_SC_) [82,83]. Currently, the most widely investigated tandem PSCs include perovskite/silicon, perovskite/perovskite, and perovskite/organic configurations [84].

### 2.2. Challenges of PSCs

Despite the significant rapid progress achieved in power conversion efficiencies, PSCs still face challenges that hinder their commercialization and practical application [85,86]. Foremost among these is the issue of operational stability, as devices degrade under the combined influence of moisture, oxygen, heat, and electrical bias, primarily due to the intrinsic hygroscopicity and ionic nature of the halide perovskite materials, necessitating effective encapsulation and isolation strategies to extend device lifetime [87,88,89]. Furthermore, finding suitable low-temperature synthesis methods is particularly critical, as the perovskite layer and certain transport layer materials are highly sensitive to elevated temperatures. Their fabrication is thus constrained by thermal stability, which limits large-scale production and the integration of flexible devices [26,32,72,90,91,92]. In addition, thickness control directly influences the optoelectronic performance of devices, where it remains difficult to achieve a balance between light transmittance and carrier concentration [28,93]. Insufficient defect passivation continues to be a bottleneck for performance enhancement, as such defects not only accelerate non-radiative recombination but also undermine long-term device stability [68,89]. Furthermore, tandem solar cells impose stricter requirements on process compatibility and overall stability [84,91,94,95,96]. Moreover, lead toxicity remains a critical environmental and health concern, driving the search for less hazardous but often less efficient lead-free alternatives. Scalability also presents a significant hurdle; the fabrication of high-quality, reproducible, pinhole-free perovskite films over large areas with uniform coverage—essential for module production—proven difficult to be achieved with laboratory-scale techniques. Finally, the development of stable, selective, and cost-effective charge transport layers that do not compromise the stability or performance of the final device is an ongoing materials challenge. These challenges and their interlinked issues—spanning stability, interface engineering, device architecture, encapsulation, toxicity, scalability and material synthesis—jointly constrain the further advancement of PSCs. Addressing them will require multidimensional optimization and technological breakthroughs to ensure the future viability of perovskite photovoltaics.

## 3. Fundamentals of ALD

### 3.1. Basic Principles of ALD and Its Variants

Atomic layer deposition (ALD), a subclass of chemical vapor deposition (CVD), was first discovered in the Soviet Union in the 1960s [97]. Since then, it has attracted widespread attention as a promising thin-film deposition technique [98,99,100,101,102]. ALD enables the deposition of films with precise thickness control, excellent uniformity, and high density at relatively low temperatures (typically below 350 °C). Moreover, ALD enables atomic-scale precision in depositing conformal thin films even on complex and large-area surfaces, without inducing damage to the underlying substrates [27,103,104,105], making it particularly well-suited for PSCs, which often incorporate thermally unstable structures.

**Figure 2:**
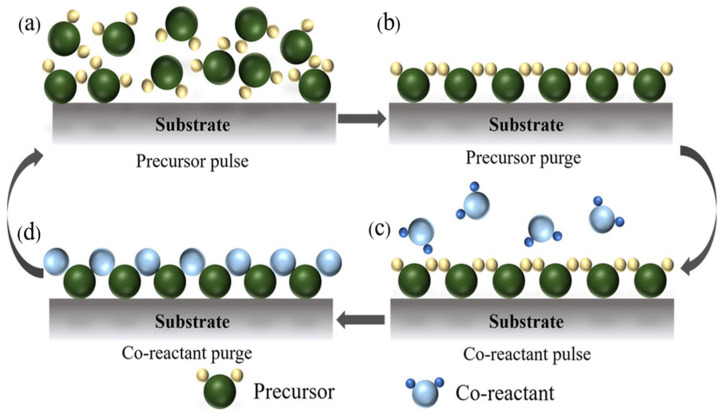
Schematic illustration of the ALD process. (**a**) Precursor adsorption on substrate active sites. (**b**) Purging of unreacted precursor molecules. (**c**) Oxidant reaction with the adsorbed precursor. (**d**) Purging of excess oxidant and by-products. Reprinted with permission from Ref. [96] Copyright 2022 Wiley-VCH GmbH.

The ALD process is based on self-limiting gas–solid surface reactions, typically consisting of two sequential “half-reactions,” as illustrated in Figure 2. In the first step, precursor gases are pulsed into the reaction chamber, where they react with functional groups on the substrate surface, forming a chemisorbed monolayer. Subsequently, an inert gas (e.g., nitrogen or argon) is used to purge unreacted precursors and byproducts. In the next step, a second precursor is introduced, which reacts with the surface to form the desired monolayer, thereby completing one cycle and regenerating the surface functional groups for the next cycle. Each deposition cycle produces a sub-monolayer of material, with the thickness determined by counting the number of reaction cycles. By controlling the number of cycles, film thickness can be tuned with atomic-level precision [30,106,107,108,109].

To address the low deposition rate of conventional ALD, a variant of ALD called Spatial Atomic Layer Deposition (S-ALD) has been developed [47,52,110]. This advanced thin-film fabrication technique retains the fundamental advantages of conventional ALD—such as exceptional conformality, sub-nanometer thickness control, and high-quality, pinhole-free growth—while overcoming its primary limitation [41,48,49]. This is achieved by replacing the temporal separation of precursors (using sequential pulses and vacuum purges in a static chamber) with a spatial separation, achieved through the physical movement of the substrate and gas zone isolation [47,48,50].

In a typical S-ALD system, precursor gases are continuously supplied to separate zones on a reactor head, which are isolated by inert gas curtains [41,47,48,50,111]. As the substrate moves relative to this head, it is sequentially exposed to each zone, enabling self-limiting surface reactions. This completes a deposition cycle in a mere fraction of a second without the need for vacuum purge steps. Consequently, S-ALD offers dramatically higher growth rates, often 10 to 100 times faster than conventional ALD. Each complete pass under the reactor head constitutes one ALD cycle, depositing a single atomic layer [47,48,53].

**Figure 3:**
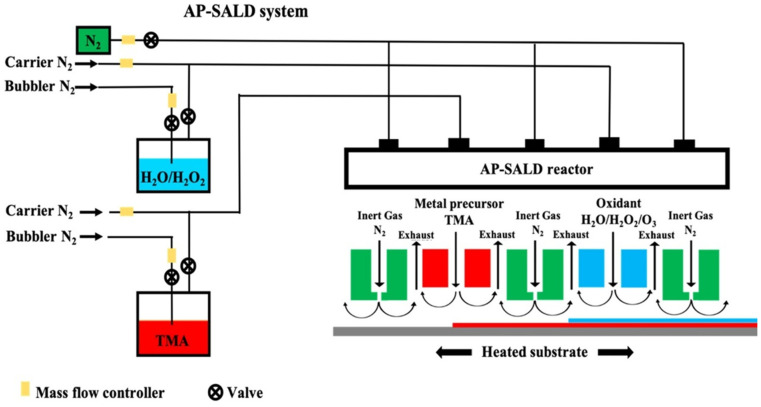
Scheme of simultaneous metal precursor and oxidant injection in AP-SALD reactor, where the precursor half-reaction zones are separated by inert gas curtains. By moving the substrate horizontally underneath the reactor, two half reactions take place sequentially on the substrate. Reprinted from Ref. [114].

S-ALD reactors can be designed to operate under low vacuum or at atmospheric pressure. The variant operating at atmospheric pressure is called Atmospheric-Pressure Spatial ALD (AP-SALD) [110,112,113]. Figure 3 shows a schematic diagram of a typical AP-SALD system [114]. This approach eliminates the need for expensive vacuum pumps and chambers, a major advantage for reducing costs and improving scalability, particularly for roll-to-roll (R2R) and large-area substrate processing [43,48,115]. These attributes make S-ALD a transformative manufacturing platform for applications ranging from barrier coatings for flexible packaging to the high-throughput production of next-generation optoelectronic devices, such as perovskite solar cells and OLED displays [47,116,117,118,119,120].

### 3.2. Advantages of ALD

**Figure 4:**
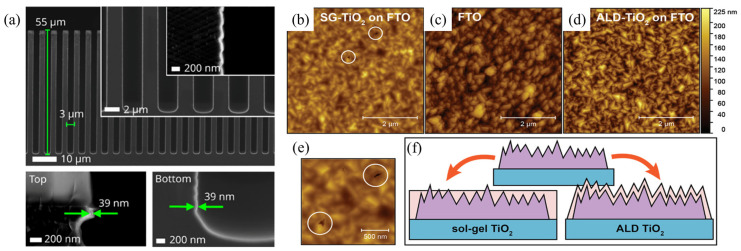
(**a**) Cross-section SEM micrographs of AlN deposited added in trenches with an 18:1 aspect ratio. Conformal deposition with a step coverage (SC) of 1 was achieved after 500 ALD cycles, resulting in a uniform film thickness of 39 nm at both top and bottom of the trench structures. Reprinted from Ref. [125] Atomic force micrographs of (**b**) an FTO substrate with TiO_2_ deposited by SG spin coating (the white circles underline the presence of deep pinholes), (**c**) a bare FTO substrate, (**d**) an FTO substrate with TiO_2_ deposited by 500 cycles of the ALD, (**e**) a magnified view of the micrograph in (**b**), and (**f**) a schematic of the SG and ALD-covered cathodes explaining the larger amount of shorted devices. The vertical scale is the same for all the AFM micrographs. Reprinted from Ref. [124].

Compared with conventional thin-film fabrication techniques such as chemical vapor deposition (CVD), sol–gel processing, spin-coating, and spray pyrolysis, ALD offers significant advantages in the fabrication of highly pure, dense, almost pinhole-free, and conformal thin films with precise control of the film thickness and composition at the atomic scale, making it essential for developing high-performance devices, including PSCs (Table 1). For example, Wu et al. [121] reported that devices employing ALD-TiO_2_ exhibited a much lower density of nanoscale pinholes than those based on TiO_2_ layers prepared by spin-coating and spray pyrolysis, leading to higher shunt resistance and substantially improved power conversion efficiency (PCE). ALD enables conformal coverage on complex morphologies, rough substrates, and high-aspect-ratio nanostructures (e.g., nanotubes, nanowires, porous surfaces and trenches) (Figure 4a), thereby ensuring complete and continuous interfacial contact between layers and greatly reducing interfacial defects [27,30,122,123]. In addition, Sukharevska et al. [124] demonstrated that TiO_2_ films prepared by sol–gel processing often contain pinholes (Figure 4b), since spin-coated sols cannot always cover the highest peaks of rough FTO surfaces. Such defects may account for the frequent short-circuiting of devices. In contrast, ALD-grown TiO_2_ films are smooth and pinhole-free (Figure 4d), which exerts a positive impact on device performance and stability.

**Table 1:** Common Thin-Film Deposition Methods Used in PSCs.

Method	Application	Compatibility with Perovskites	Characteristics	Refs.
Sol–gel method	TiO_2_, ZnO, NiO, etc.	Pinholes can induce leakage current and oxygen vacancies; surface roughness inhomogeneity may lead to short circuits	Simple operation; highly sensitive to environmental conditions; poor reproducibility	[28,29,90,126,127,128,129,130]
Sputtering	NiO, ZnO, ITO, SnO_2_, etc.	Potential damage to the perovskite layer due to ion bombardment	Uniform deposition; strong film adhesion; relatively high cost	[103,131,132,133,134,135,136,137]
Thermal Evaporation	MoO_x_, CuI, CdS, CO, etc.	Vacuum-induced thermal stress can cause severe degradation of organic halides; annealing temperature of materials is limited	Fast deposition rate; suitable for low-melting-point materials; difficult to deposit high-melting-point oxides; good film uniformity	[87,103,135,138,139,140,141,142]
Chemical Vapor Deposition	CuO, TiO_2_, ZnO, etc.	High deposition temperature is unfavorable for thermally sensitive perovskite materials	Low cost, high scalability, and fast deposition rate with good step coverage; however, the high deposition temperature may impair other solar cell components, and introduce impurities during the process	[103,143,144,145,146,147]
Atomic Layer Deposition	TiO_2_, SnO_2_, Al_2_O_3_, VO_x_, etc.	Slow deposition rate; vacuum environment increases cost	Enables conformal growth with uniform and pinhole-free films; precise thickness control via self-limiting reactions; allows low-temperature synthesis compatible with thermally unstable PSCs; uniform interfaces facilitate carrier dynamics regulation	[28,92,93,148,149]
Spray Pyrolysis	TiO_2_, NiO, SnO_2_, CuCrO_2_, CuI, etc.	High temperature is unfavorable for thermally sensitive perovskite materials; prone to pinhole formation, leading to carrier recombination and short circuits	Low cost and simple operation; suitable for large-scale applications, but process parameters are difficult to control precisely; pinholes may remain on the film surface	[28,150,151,152,153,154,155]
Spin Coating	SnO_2_, ZnO, CuI, etc.	Requires high annealing temperature, which is unfavorable for thermally sensitive perovskite materials; irregular surface prone to pinhole formation, leading to carrier recombination and short circuits	Not suitable for large-area deposition; limited control over film thickness and uniformity on rough surfaces; high annealing temperature required	[20,92,129,132,138,156,157,158]

Another critical advantage of ALD lies in its low-temperature processing capability, which is particularly suited for thermally unstable perovskite materials and flexible polymer substrates, thereby avoiding the decomposition or interfacial reorganization associated with high-temperature treatments [10,72,91,92].

The variant of ALD, namely AP-SALD, retains the core benefits of ALD, such as excellent conformality on complex structures, precise thickness control via cycle counting, and high-quality, pinhole-free films [43,47,48,51,52,58,110,112,113]. Additionally, it offers the advantage of a dramatically increased deposition speed compared to conventional ALD. By eliminating the time-consuming vacuum purge steps, deposition rates can be 10 to 100 times faster [48,58,116,159]. This makes it compatible with roll-to-roll (R2R) manufacturing for high-throughput industrial production [43,160,161,162]. Furthermore, operation at atmospheric pressure removes the need for high-vacuum pumps and chambers, significantly reducing the system’s initial cost, energy consumption, and maintenance requirements [48,58,113]. The spatial approach is also inherently easier to scale up for large-area substrates than vacuum-based systems [47,48,58,115]. AP-SALD is particularly promising for industries that require high-throughput, low-cost, and large-area coating of high-quality thin films [47,48,50]. It is a transformative manufacturing-oriented version of ALD that sacrifices the ultra-high purity of vacuum environments for the immense benefits of speed, cost, and scalability, making it a key enabler for the commercial adoption of ALD in mass production.

Beyond metal oxides such as TiO_2_ [32,72], SnO_2_ [26,92,163,164,165], and Al_2_O_3_ [28], ALD has been successfully extended to metals, [166,167] nitrides, [168,169] and sulfides [170,171], thereby greatly broadening its applicability to solar cells, electronic devices, gas sensors, and energy storage systems. Moreover, ALD holds promise for the synthesis of novel PSC materials. For instance, most hole transport layers (HTLs) currently used in PSCs are organic polymers, which are relatively expensive and thus limit large-scale applications [172,173]. While TiO_2_ has been widely employed as an electron transport layer (ETL) in photovoltaics [32], it can also serve as an HTL when its work function is sufficiently high. Tan et al. [174] demonstrated that through a super-cycled ALD process, TiO_2_ could be alloyed with IrO_x_, effectively transforming it into a functional HTL, thereby reducing the overall fabrication cost of PSCs.

With its precise thickness controllability, excellent uniformity and conformality, wide low-temperature processing window, and strong substrate compatibility, ALD is increasingly regarded as a core enabling technology for next-generation electronic and optoelectronic devices [175]. Its application in PSC interface engineering is expected to offer new strategies for addressing key challenges related to efficiency, stability, and scalability.

## 4. ALD Applications in PSCs

### 4.1. Low-Temperature Synthesis

In PSC fabrication, low-temperature processing provides an effective approach to addressing the thermal sensitivity of constituent materials [10,92]. Although traditional high-temperature treatments can improve crystallinity, they often damage the perovskite and organic layers, thereby limiting device design. Temperature Limits for thermally unstable layers in perovskite solar cells are in Table 2. Owing to its controllable low-temperature growth and superior interfacial coverage, ALD not only enhances charge collection and blocking properties in various transport layers but also demonstrates considerable potential for large-scale manufacturing of PSCs.

**Figure 5:**
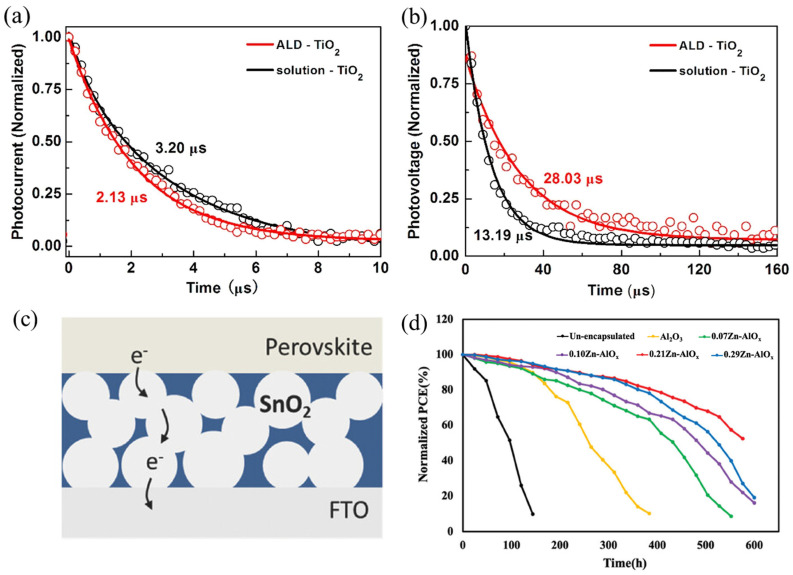
(**a**) TPC and (**b**) TPV results of PSCs with ALD-TiO_2_ and solution-TiO_2_ ETLs, respectively. The symbols represent the experimental data and the solid lines indicate the fitting curves. Reprinted with permission from Ref. [32]. Copyright 2019 International Solar Energy Society. Published by Elsevier Ltd. All rights reserved. (**c**) The proposed structure of the low-temperature-processed ALD SnO_2_ film. The SnO_2_ particles are self-passivated by the residual precursor depicted as a blue region. Reprinted from Ref. [26]. (**d**) Normalized PCE versus time. Reprinted from Ref. [10].

The advantages of low-temperature ALD were first demonstrated in titanium dioxide (TiO_2_) [26,72,186]. Owing to its favorable band alignment with perovskite conduction bands and low processing cost, TiO_2_ has been widely adopted as an ETL. However, conventionally prepared TiO_2_ often requires high-temperature annealing (>450 °C) and exhibits limited interfacial charge collection ability. To address this, Chen et al. [32] fabricated TiO_2_ ETLs using low-temperature ALD (150 °C). Compared with solution-processed ETLs (PCE = 18.64%, FF = 76.1397%, V_OC_ = 1.0356 V), the ALD-derived films yielded higher PCE (19.45%), improved fill factor (78.1532%) and open-circuit voltage (1.0829 V), along with superior photo-stability and reproducibility. From Figure 5a, it is evident that PSCs employing ALD-TiO_2_ ETLs exhibit a faster decay in transient photocurrent (TPC), indicating more efficient charge extraction and transport compared to their counterparts with solution-processed TiO_2_ ETLs. Meanwhile, the transient photovoltage (TPV) reveals that the charge recombination lifetime of PSCs with ALD-TiO_2_ ETLs is substantially longer, confirming that TiO_2_ prepared by ALD effectively suppresses charge recombination in PSCs (Figure 5b). Collectively, these results underscore the potential of low-temperature ALD-TiO_2_ ETLs in enabling highly efficient PSCs and advancing the development of flexible device architectures.

Besides TiO_2_, low-temperature ALD has also shown promise in depositing tin dioxide (SnO_2_) films, which features higher electron mobility, low thermal processing requirements, and excellent optical transparency, making it an ideal candidate to replace TiO_2_ [165,187]. Lee et al. [26] reported that devices based on SnO_2_ films fabricated by wet chemical methods followed by high-temperature annealing (300 °C) suffered significant reductions in V_OC_ and PCE. This was attributed to reduced self-passivated SnOCl_2_, which weakened the hole-blocking capacity of the SnO_2_ ETL. Furthermore, they employed a low-temperature ALD process (100 °C deposition) and compared samples post-annealed at 180 °C and 300 °C. The results revealed that SnO_2_ films annealed at lower temperatures exhibited reduced series resistance and properly controlled surface passivation, the corresponding structure of which is presumed to be illustrated in Figure 5c, thereby enhancing device efficiency. These findings indicate that low-temperature ALD is a key strategy for achieving high-quality SnO_2_ ETLs and optimizing device performance.

Low-temperature ALD also enables efficient device encapsulation without damaging the perovskite layer. Various deposition methods have been explored for preparing thin-film encapsulation (TFE) in PSCs; however, conventional approaches often require high energy input or elevated temperatures, which can degrade perovskite materials during processing. Asgarimoghaddam et al. [10] employed an AP-SALD system to directly deposit a 60 nm thick Zn-AlO_x_ encapsulation layer onto PSCs at 130 °C, without damaging the thermally sensitive underlying perovskite or organic layers. By tuning the Zn-AlO_x_ composition, they found that increasing the Zn/Al ratio to 0.21 reduced the water vapor transmission rate (WVTR) to 1.3 × 10^−4^ g/m^2^/day, as measured by a Ca test at 65 °C and 85% RH. All samples with different Zn/Al ratios exhibited WVTR values on the order of 10^−4^ g/m^2^/day. It was found that the p–i–n PSCs encapsulated with a 0.21Zn-AlO_x_ TFE layer retained 80% of its initial PCE after 384 h of exposure at 65 °C and 85% relative humidity, while the unencapsulated device remained stable for only 52 h (Figure 5d).

Furthermore, the combination of low-temperature ALD with the highly reactive oxygen sources shows great potential in high-performance optoelectronic devices. Ren et al. [91] demonstrated that by employing hydrogen peroxide (H_2_O_2_) as the oxygen source during ALD, the deposition temperature could be reduced to as low as 50 °C. Such low-temperature growth enabled conformal coating of substrates and the formation of pinhole-free films with high optical transmittance, excellent electron extraction, and large-area uniformity. Compared with the sample synthesized at 100 °C, the SnO_2_ film deposited at 50 °C exhibited clearer microstructural features in the SEM images, indicating higher electrical conductivity. In addition, the SnO_2_ grown at 50 °C showed a higher growth rate, which was likely attributed to increased steric hindrance and the thermal decomposition of H_2_O_2_ into H_2_O and O_2_ at elevated temperatures. The device based on 50 °C ALD-SnO_2_ achieved a PCE of 20.70% in single-junction PSCs, whereas the 100 °C counterpart exhibited only 17.35%.

### 4.2. Thickness and Composition Control

The thickness and composition of PSC films critically affect light transmittance, carrier concentration, and interfacial recombination processes [28,93]. Owing to its atomic-level thickness controllability and precise compositional tunability, ALD not only optimizes the electrical and interfacial properties of ultrathin layers such as Al_2_O_3_ and NiO_x_, but also improves the conductivity and stability of ETLs such as SnO_2_ via elemental doping, thereby significantly enhancing the efficiency and reliability of PSCs.

**Figure 6:**
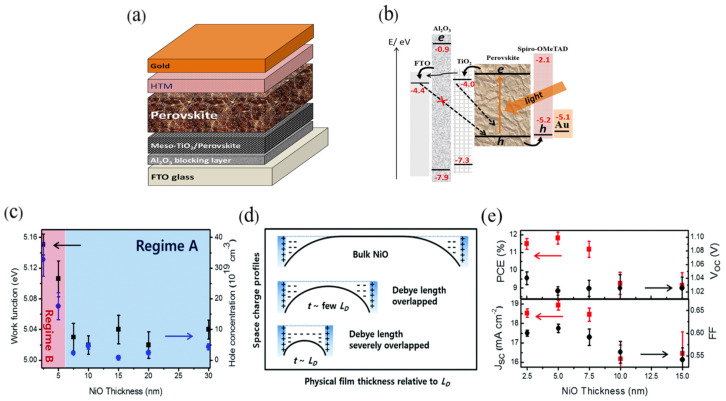
(**a**) Perovskite solar cell structure. (**b**) Charge transfer and transport pathways in PSCs with Al_2_O_3_ as blocking underlayer. Reprinted with permission from Ref. [28]. Copyright 2017 Wiley-VCH Verlag GmbH & Co. KGaA, Weinheim. (**c**) Plot of the work function and hole concentration vs. the film thickness of NiO measured by electrochemical Mott–Schottky analysis at selected frequencies of 8 to 20 kHz in the electrolyte of pH = 12. Black squares and blue circles indicate the work function and doping density, respectively. NiO films thicker than 7.5 nm (Regime A) show similar work function and hole concentration, while thinner than 7.5 nm (Regime B) show an increase in the function of thickness. (**d**) Schematic images of physical films’ thickness relative to the Debye length (LD) vs. space charge profiles. When the thickness of NiO films was large enough compared to the LD, the NiO films were bulk-like, thus insulating ones as shown in the inset (top). Once the films’ thicknesses were thin enough, the LD started to overlap, and thus the apparent work function and hole concentration are increased (schematic in middle). Even in thinner films, LD is severely overlapped (schematic in the bottom). (**e**) Plot (top) of the power conversion efficiency (PCE, %) and open circuit voltage (V_OC_, V) vs. the thickness of NiO films (2.5, 5.0, 7.5, 10.0 and 15.0 nm in thickness). Plot (bottom) of the photocurrent density (J_SC_, mA cm^−2^) and fill factor (FF) vs. the thickness of NiO films (2.5, 5.0, 7.5, 10.0 and 15.0 nm in thickness). Reprinted with permission from Ref. [93] Copyright 2016 RSC Pub.

The film thickness of ALD-synthesized layers is highly tunable. Zhang et al. [28] first introduced ALD-grown Al_2_O_3_ as an underlayer in PSCs (Figure 6a). By varying the thickness of the Al_2_O_3_ underlayer, they investigated changes in interfacial charge recombination, photovoltaic performance, and the possibility of electron tunneling through the Al_2_O_3_ layer. Figure 6b presents a schematic illustration of the charge transfer and transport processes in PSCs. When 50 ALD cycles (corresponding to ~5 nm) were deposited, the PSC achieved a PCE of 16.2%, which was a 43% improvement compared with the 11.0% PCE of PSCs without the underlayer. The dense, pinhole-free Al_2_O_3_ film suppressed electron recombination at the FTO/perovskite interface, thereby enabling high-performance PSCs.

ALD also excels in the precise control of ultrathin films. Nickel oxide (NiO_x_) is an excellent HTL with high optical transparency and chemical stability; however, undoped NiO suffers from high resistivity. Compared with conventionally prepared NiO films, ALD-grown ultrathin NiO films allow more accurate thickness control, yielding distinct conductive properties. Seo et al. [93] demonstrated that ALD-deposited NiO films with a thickness of ~1–2 nm—close to the Debye length—exhibited significantly increased work function and hole concentration (Figure 6). NiO films with a thickness of 5–7.5 nm, corresponding to several times the Debye length, exhibit sufficient conductivity due to the overlap of space charge regions. Devices incorporating such films achieved a maximum PCE of 16.40% with negligible hysteresis, thereby improving PSC photovoltaic performance. Importantly, NiO films prepared via ALD can achieve higher conductivity without sacrificing optical transparency.

**Figure 7:**
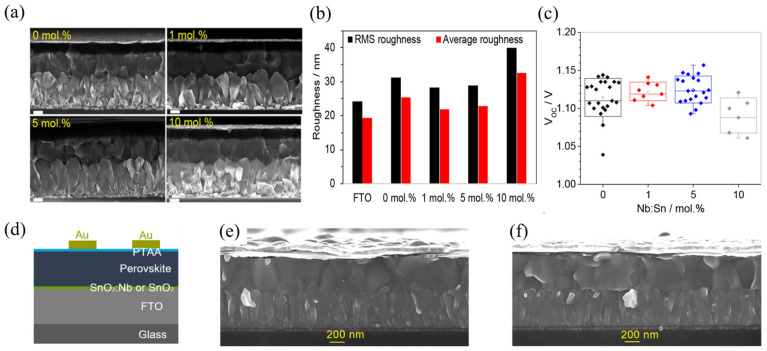
Nb-doped SnO_2_-based ETLs prepared by CBD with 0, 1, 5, and 10 mol% Nb doping: (**a**) the cross-sectional SEM images of full devices with FTO-coated glass/SnO_2_-based ESL/perovskite/spiro-OMeTAD/gold configuration, the scale bars are 200 nm in SEM images. (**b**) Root-mean-square (RMS) and average roughness values of SnO_2_-based ESLs compared with the FTO substrate. (**c**) V_OC_ statistics of planar perovskite solar cells fabricated using Nb-doped SnO_2_-based ESLs. Reprinted with permission from Ref. [188]. Copyright 2018 American Chemical Society. Nb-doped SnO_2_-based ETLs prepared by ALD: (**d**) Schematic of the n–i–p PSC architecture, and cross-sectional SEM images of the completed devices for (**e**) S_0_ and (**f**) O_55_ (O/S describing the precursor introduction sequence and the numbers representing the SnO_2_/Nb_2_O_5_ cycle ratio. S_0_ that is a device with the pristine SnO_2_ ETL was used as a reference). Reprinted with permission from Ref. [31]. Copyright 2025 The Authors. Published by American Chemical Society.

In addition, ALD enables precise compositional control without compromising surface roughness. Doping strategies are particularly effective in enhancing conductivity and electron mobility. For example, the electrical performance of SnO_2_, a widely used ETL material, is strongly influenced by tin interstitials and oxygen vacancies [163], which often require optimization. Halvani et al. [188] attempted Nb doping in SnO_2_ using chemical bath deposition (CBD). However, when the Nb content exceeded a certain threshold, the device V_OC_ decreased presumably due to increased ETL roughness, leading to poor surface coverage and incomplete contact with the perovskite layer (Figure 7a–c). To overcome these limitations, Gesesse et al. [31] employed ALD to fabricate relatively smooth Nb-doped SnO_2_ films (Figure 7e,f). By adjusting the ALD cycle ratio of SnO_2_:Nb, they were able to fine-tune the optoelectronic properties of the resulting PSCs.

### 4.3. Defect Passivation

In PSCs, defects at both the bulk and interfacial levels are widely regarded as one of the primary causes of charge recombination, performance degradation, and reduced stability [89]. ALD enables the introduction of dense passivation layers at interfaces or surfaces, which can effectively reduce defect-state density, suppress non-radiative recombination, and thereby significantly enhance the efficiency and long-term stability of PSCs.

**Figure 8:**
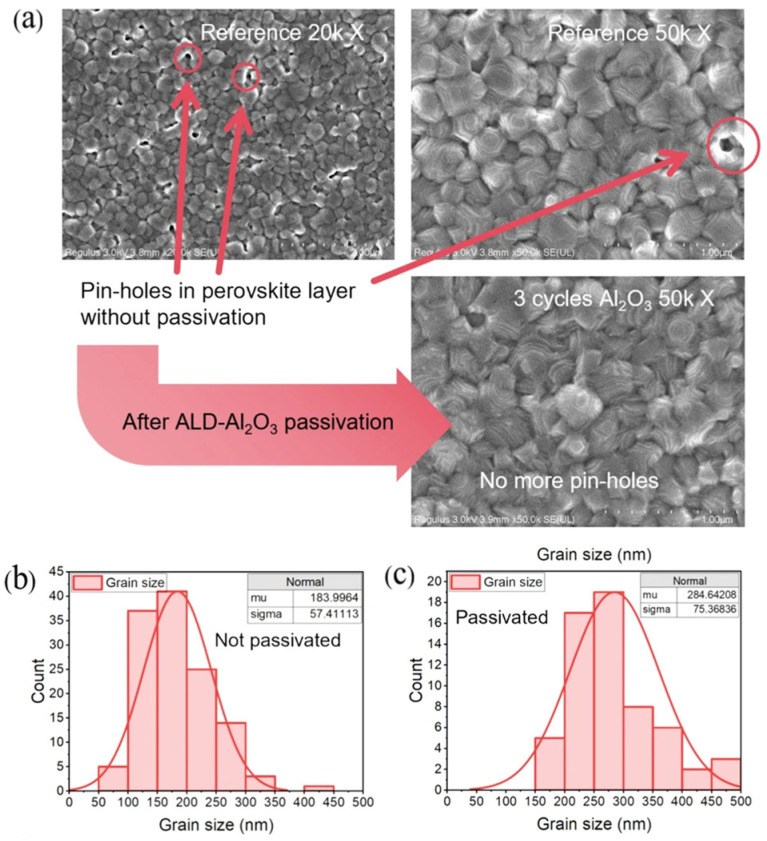
(**a**) Surface SEM images of the perovskite layer of the reference group and 4 cycles ALD-Al_2_O_3_ group at 20,000 and 50,000× magnification. (**b**,**c**) Grain size distribution diagram of the perovskite layer based on 50,000× SEM images in (**a**). Reprinted with permission from Ref. [189]. Copyright 2025, Youke Publishing Co., Ltd.

Conventional thin-film deposition methods often result in defect-rich perovskite interfaces, where photogenerated carriers recombine rapidly, leading to sharp declines in device performance. To tackle these challenges, Xiao et al. [189] introduced a nanoscale Al_2_O_3_ layer at the perovskite/NiO_x_ interface using ALD. This strategy mitigated the high density of trap states in NiO films, effectively suppressed carrier recombination, and improved valence band alignment between the HTL and perovskite, thereby facilitating hole transport. Furthermore, they observed that increasing the number of ALD cycles promoted the growth of better self-assembled monolayers, which enlarged perovskite grain size, reduced pinholes (Figure 8a), and lowered overall Al_2_O_3_ roughness (Figure 8b,c).

**Figure 9:**
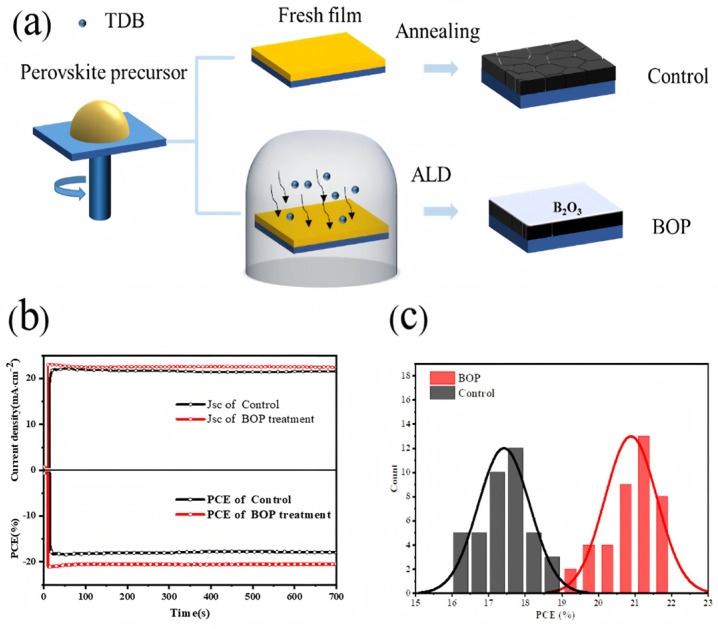
(**a**) Schematic of the formation process for the control and B_2_O_3_-treated perovskite films. (**b**) Steady-state output PCE (control device with a bias voltage of 0.81 V, B_2_O_3_ treatment device with a bias voltage of 0.91 V). (**c**) PCE distribution of the control and BOP PSC. Reprinted with permission from Ref. [89]. Copyright 2024 American Chemical Society.

Similarly, Jia et al. [89] deposited ultrathin boron oxide (B_2_O_3_) layers (3 nm) on the surface of perovskite films via ALD (Figure 9a). The B_2_O_3_-passivated devices (denoted as the BOP group) exhibited larger grain sizes compared with control devices, thereby reducing defect density and suppressing non-radiative recombination. The strong electron-donating side oxygen atoms in B_2_O_3_ molecules effectively interacted with positively charged defects in perovskites, thereby enhancing device performance. Perovskite degradation typically generates Pb^0^ defects, which contribute to non-radiative recombination [190]. ALD-deposited B_2_O_3_ also interacted with undercoordinated Pb^2+^, suppressing perovskite degradation and reducing Pb^0^ formation. As shown in Figure 9b,c, BOP devices achieved a stabilized PCE of 20.48% and a stabilized J_SC_ of 22.50 mA·cm^−2^, significantly higher than control devices (17.83% PCE and 21.49 mA·cm^−2^ J_SC_), while the hysteresis index was reduced by 53.6%. Moreover, the B_2_O_3_ layer accelerated interfacial charge transfer at the perovskite/HTL interface, improving the operational stability of unencapsulated PSCs.

### 4.4. Isolation and Encapsulation

Perovskite materials are extremely sensitive to humidity [10], temperature, and light exposure. Their poor environmental stability has become a central bottleneck limiting the operational lifetime and practical deployment of PSCs [164,189,191,192]. In this context, dense, pinhole-free oxide films prepared by ALD, with excellent gas and moisture barrier properties, are regarded as a promising encapsulation strategy to enhance long-term device stability [193].

**Figure 10:**
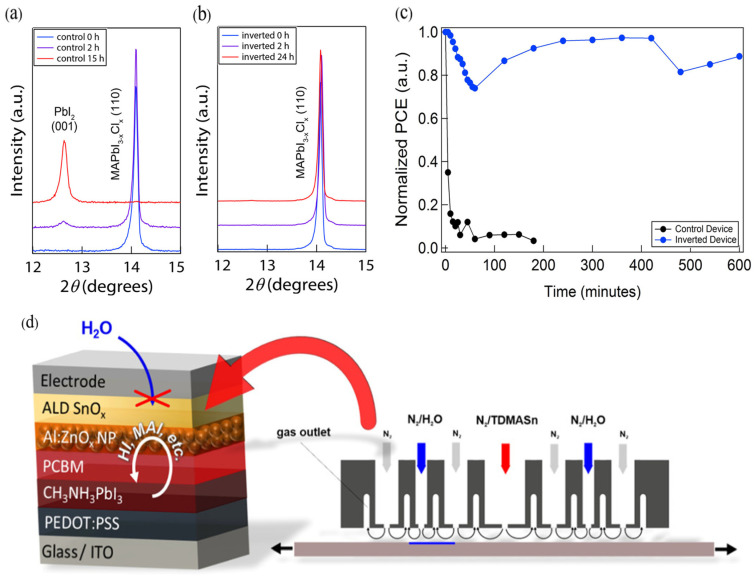
Time-dependent X-ray diffraction spectra of (**a**) control planar perovskite device and (**b**) inverted perovskite device before and after thermal soaking at 100 °C. (**c**) Stability of control and inverted perovskite devices without additional encapsulation upon thermal soaking at 100 °C extracted from I–V measurements. Reprinted with permission from Ref. [186]. Copyright 2016 American Chemical Society. (**d**) p–i–n solar cell stack used in this work and schematic of the spatial ALD assembly. Reprinted with permission from Ref. [191]. Copyright 2018 American Chemical Society.

ALD is capable of depositing continuous and conformal encapsulation layers on complex surfaces. For instance, researchers [186] introduced an ALD-grown amorphous TiO_2_ interlayer in PSCs, forming a uniform pinhole-free coverage that not only suppressed the penetration of moisture and oxygen but also prevented the escape of methylammonium iodide (MAI) under thermal stress, thereby effectively mitigating thermal degradation of the perovskite absorber. This strategy significantly enhanced the device stability, as evidenced by the XRD patterns and J-V curves after prolonged thermal soaking at 100 °C (Figure 10a–c). Moreover, the conformal growth inherent to ALD ensured complete and continuous coverage of amorphous TiO_2_ even on highly complex absorber topographies, offering distinct advantages over conventional deposition techniques.

ALD also enables the fabrication of improved barrier layers. Conventionally solution-processed SnO_x_ films contain pinholes, rendering them ineffective as diffusion barriers. Behrendt et al. [194] fabricated dense, conformal SnO_x_ films by low-temperature ALD, achieving excellent water vapor transmission rates (WVTR as low as 10^−6^ g·m^−2^·day^−1^) and maintaining stable performance for over 50 days under damp-heat conditions. Hoffmann et al. [191] were the first to apply spatial ALD (S-ALD) to deposit impermeable SnO_x_ ETLs, which could be incorporated into PSC stacks without affecting photovoltaic performance (Figure 10d), while simultaneously preventing moisture ingress and suppressing decomposition of the perovskite active layer.

**Figure 11:**
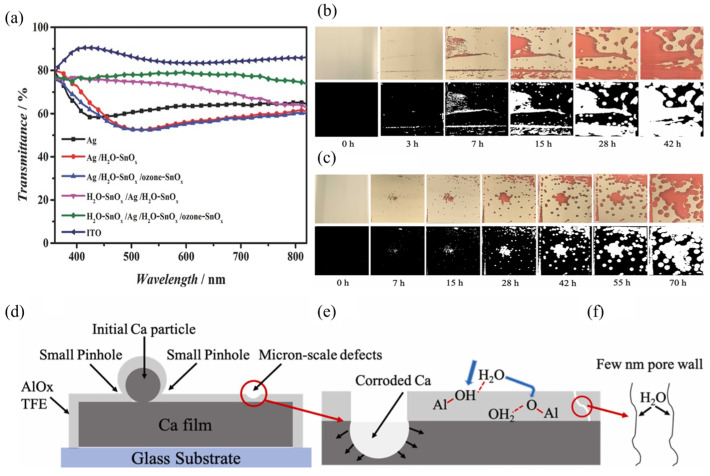
(**a**) Optical transmission spectra of different Ag and SnO_x_ based electrodes on glass. Reprinted with permission from Ref. [164]. Copyright 2017 WILEY-VCH Verlag GmbH & Co. KGaA, Weinheim. Real pictures and high-contrast black-and-white images of 1.8 cm × 1.8 cm Ca films encapsulated with (**b**) AlO_x_ and (**c**) N-AlO_x_, barrier films with a thickness of 60 nm after storage in 25 °C and 55% RH conditions for increasing time intervals. Schematic of (**d**) pinholes and micron scale defects on AlO_x_ thin film encapsulation layer, (**e**) Ca corrosion and the percolation paths formed progressively with time resulting from H_2_O permeating along chains of chemical defect clusters like -OH defects, and (**f**) surface diffusion of water through pore walls. Reprinted with permission from Ref. [119]. Copyright 2024 The Authors. Published by Elsevier Ltd.

In addition, SnO_x_ films have been shown to effectively protect sensitive metal electrodes from corrosion caused by halide species originating from perovskites, thereby delaying electrode degradation. Hu et al. [164] observed that the conductivity of an ultrathin Ag layer completely vanished after a perovskite film was deposited on it; they attributed this loss of conductivity to chemical reactions between Ag and halide species such as methylammonium iodide (MAI). To prevent such corrosion, they introduced an H_2_O-SnO_x_ protective layer on top of the Ag film, which maintained a nearly constant sheet resistance (Rsh). The optical transmittance spectra of the electrodes were consistent with the Rsh results (Figure 11a). For samples where Ag was directly deposited on glass, the overall transmittance was lower and exhibited a characteristic V-shaped spectrum, which was attributed to the island-like growth of silver. After coating with a thin H_2_O-SnO_x_ layer, the transmittance minimum red-shifted due to an increase in the dielectric constant around the silver particles, resulting in a plasmonic resonance shift toward longer wavelengths. In contrast, when Ag was deposited on top of the H_2_O-SnO_x_ layer, no V-shape appeared in the transmittance spectrum, indicating significantly improved wettability and a more percolated Ag film. These findings demonstrate that the H_2_O-SnO_x_ layer effectively protects the Ag electrode from corrosion by perovskite precursors.

Beyond ALD and S-ALD, atmospheric-pressure spatial ALD (AP-SALD) has also been widely applied. Asgarimoghaddam et al. [119] employed AP-SALD to deposit nitrogen-doped alumina (N-AlO_x_) thin-film encapsulations (TFEs). In conventional ALD, lowering the processing temperature often deteriorates the barrier performance of AlO_x_ TFEs due to reduced film density and the presence of carbon-related impurities and hydroxyl groups. In contrast, AP-SALD enables deposition at sufficiently high temperatures (130 °C) to achieve dense barrier films, while maintaining a high growth rate that prevents thermal damage to the perovskite layer. By tuning the nitrogen doping level through AP-SALD, the minimum WVTR of 1.34 × 10^−5^ g·m^−2^·day^−1^ (at 25 °C and 55% RH) was achieved at 0.28% nitrogen content. As shown in Figure 11b,c, the stability of N-AlO_x_ was significantly enhanced compared to undoped AlO_x_. Numerous circular spots observed in Figure 11b were identified as oxidation regions, likely caused by pinhole defects in the AlO_x_ coating. These oxidation regions expanded over time, providing diffusion pathways for water, as illustrated schematically in Figure 11d–f. Notably, the T_80_ lifetime of the PSCs with 0.28% N-AlO_x_ encapsulation increased from 144 h to 855 h, clearly demonstrating the potential of AP-SALD N-doped AlO_x_ in improving long-term device stability.

### 4.5. Tandem Solar Cells

Tandem solar cells, owing to their higher energy utilization and efficiency potential, are considered one of the most promising future directions in photovoltaics [195,196]. However, issues such as temperature incompatibility, insufficient interfacial stability, and poor interlayer compatibility remain major obstacles to their practical implementation. With its low-temperature processing, conformal coverage, and precise controllability, ALD provides effective solutions to these bottlenecks.

**Figure 12:**
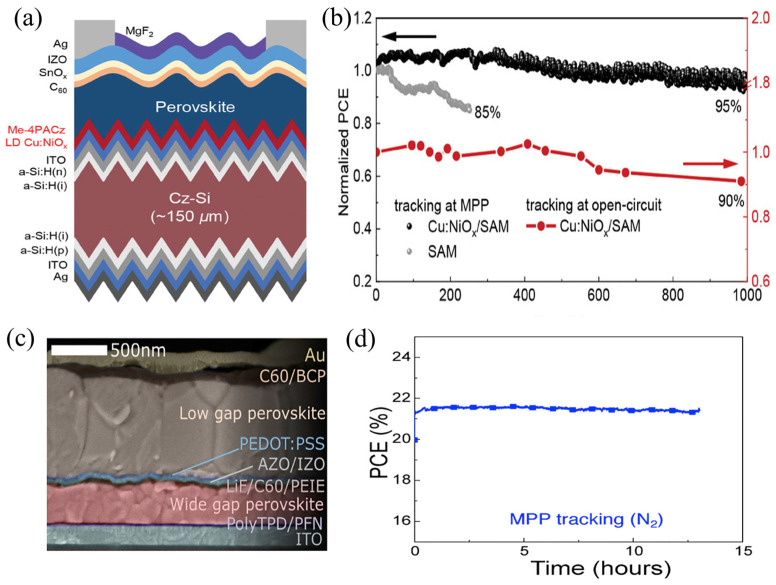
(**a**) The device structure of textured perovskite/silicon tandems using ALD Cu:NiO_x_/SAM bilayer HTL. (**b**) Long-term operational stability of encapsulated tandem solar cells. Black dots and gray dots indicate the tandems using Cu:NiO_x_/SAM and SAM HTL continuous MPP tracking under 0.8-sun infrared-enhanced white LED, respectively; red dots indicate tandems using Cu:NiO_x_/SAM HTL accelerated aging at open-circuit condition under 1-sun illumination using AAA class solar simulator. Reprinted with permission from Ref. [198]. Copyright 2024 Wiley-VCH GmbH. (**c**) Cross-sectional scanning electron micrograph of all-perovskite tandem with AZO layers. (**d**) Longer maximum power point tracking for a rigid tandem with AZO layers, under AM1.5 illumination in nitrogen for 13 h. Reprinted with permission from Ref. [199]. Copyright 2019 Elsevier Inc.

In perovskite/silicon tandem cells, the development of certain ALD-grown films, such as NiO_x_, has been restricted because post-deposition annealing typically requires temperatures above 300 °C, which exceed the thermal budget of silicon heterojunction solar cells (<200 °C) [197]. Zhu et al. [198] addressed this limitation by employing ALD to fabricate Cu-doped NiO_x_ (ALD Cu:NiO_x_) HTLs at low temperatures (Figure 12a). This approach enabled high device performance while maintaining compatibility with the thermal constraints of silicon heterojunctions. The tandem device showed significantly enhanced photovoltaic performance, and after 1000 h of maximum power point (MPP) tracking, it retained 95% of its initial efficiency, thereby demonstrating the feasibility of fabricating perovskite/silicon tandems at low temperatures (Figure 12b).

Perovskite tandem solar cells offer the advantages of low processing cost, high-throughput fabrication, and compatibility with flexible substrates [3]. However, achieving high optoelectronic performance while preventing damage to the bottom sub-cell during top-cell processing remains a key challenge. Palmstrom et al. [199] employed ALD to nucleate conformal, low-conductivity aluminum-doped zinc oxide (AZO) layers in perovskite tandem solar cells (Figure 12c). The composite layer exhibited a high sheet resistance on the order of 10–100 kΩ/sq, which effectively suppressed lateral conduction toward shunt pathways. It mitigated solvent-induced degradation of the underlying perovskite absorber during solution processing, thereby preventing damage to the bottom cell and improving both the performance and stability of perovskite tandem devices. The tandem device operated at its maximum power point under one-sun illumination for 13 h without any observable performance degradation (Figure 12d).

**Figure 13:**
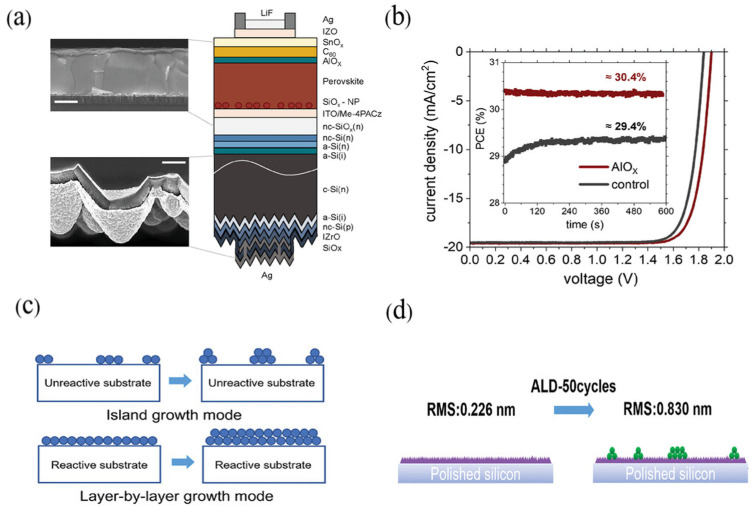
(**a**) Device structure and SEM image of monolithic two-terminal perovskite/silicon tandems utilized. Scale bar is 300 nm (top) and 1 µm (bottom), respectively. (**b**) JV curves of champion target and control devices with MPPT measurement over 10 min in the inset. Reprinted from Ref. [95]. (**c**) A schematic of island growth and layer-by-layer growth modes on different substrates. (**d**) Schematic diagram of two growth modes on PCBM and AZO substrates. Reprinted with permission from Ref. [94]. Copyright 2022 Wiley-VCH GmbH.

ALD has also been applied to modify the top cells of perovskite/silicon tandems. Artuk et al. [95] introduced an ALD-deposited ultrathin alumina (AlO_x_) layer on wide-bandgap perovskite absorbers serving as the top cell in monolithic perovskite/silicon tandems (Figure 13a). The AlO_x_ layer was thin enough to allow electron transport, yet sufficiently thick to suppress interfacial recombination, leading to an absolute increase in PCE. As shown in Figure 13b, the best-performing device achieved a stabilized efficiency of 30.4%, compared to 29.4% without AlO_x_. This improvement stemmed primarily from an increase in the product of fill factor (FF) and V_OC_, contributing approximately 50 mV to the overall gain.

ALD can be applied to activate inert substrates used in tandem PSCs. When the substrate surface does not react with ALD precursors, the growth tends to proceed in an island-like fashion (Figure 13c), resulting in pinhole-rich films that degrade device performance [200]. To overcome this, Yu et al. [94] introduced reactive sites by employing AZO with hydroxyl groups to activate the otherwise inert PCBM layer, thus facilitating the layer-by-layer growth of SnO_x_ by ALD and enabling the formation of pinhole-free SnO_x_ buffer layers (Figure 13d). This strategy effectively resolved the pinhole issue on inert surfaces. The resulting devices exhibited a PCE of 23.31%, and retained over 99% of their initial power conversion efficiency even after aging for more than 5100 h, demonstrating outstanding operational stability.

## 5. Limitations and Challenges

Despite the tremendous potential of ALD in PSCs, several challenges remain. Although ALD offers the advantage of low-temperature growth, which reduces thermal damage to heat-sensitive perovskite layers compared with conventional high-temperature treatments, certain oxidizing precursors and plasma species in plasma-assisted ALD can still induce unfavorable modifications on the perovskite surface [10,26,189,201]. To mitigate this issue, some researchers have introduced buffer or protection layers between the ALD and perovskite layers, typically prepared by spin-coating or related techniques, to prevent direct reactions with oxidants [202]. Others have developed hybrid ALD modes, alternating thermal and plasma ALD cycles, to minimize plasma-induced damage to the perovskite [201]. Although these approaches help alleviate the problem, they also increase process complexity and interfacial bonding challenges, indicating that further optimization is still required for applying ALD in PSCs.

Another major limitation lies in the high equipment cost and scalability constraints. Although uniform large-area films have been successfully fabricated using ALD in PSCs [91,92], the stringent requirements for precise control over gas flow, temperature, and pressure render ALD equipment and maintenance significantly more expensive than conventional deposition methods. In addition, the relatively slow deposition rate hinders its suitability for large-scale industrial production. In large-area device fabrication, substrate non-uniformity and potential growth defects may also lead to inconsistency in the electrical properties of the film, thereby affecting device efficiency and stability. To tackle these challenges, S-ALD [10,119] has been proposed, which can enhance deposition rates through compartmentalized reactions and improve large-area uniformity. SALD can achieve deposition rates up to 100 times faster than conventional ALD [203,204]. Although promising, S-ALD remains in an exploratory stage and is not yet ready for mature industrial deployment, as the following challenges remain to be addressed.

A significant drawback is that S-ALD tends to have higher precursor consumption compared to temporal ALD. This is because precursors often flow continuously across the substrate, even when not actively reacting. Consequently, some precursor molecules are inevitably carried away by the inert gas curtains or simply flow off the edge of the substrate without participating in a surface reaction [204]. This leads to lower precursor utilization efficiency, which is a critical economic factor for expensive precursors [205].

Furthermore, S-ALD requires a more complex reactor design to prevent gas-phase mixing, which is a primary engineering hurdle [206]. The entire principle of S-ALD relies on maintaining perfect spatial separation of the precursor gases [47,58,207]. If they mix, it causes parasitic gas-phase reactions, leading to particle formation and non-conformal, CVD-like growth [204]. Achieving this separation requires extremely precise design and machining of the gas delivery head. This makes the reactor inherently more complex than a simple vacuum chamber [208,209].

In addition, not all ALD chemistries can be easily transferred from temporal to spatial reactors. Chemistries involving extremely rapid gas-phase reactions, or those that are highly reactive with the carrier/inert gas, are more prone to mixing before reaching the substrate surface. The continuous supply of precursors can also be a challenge, particularly for solid or liquid precursors with very low vapor pressure, as maintaining a stable, consistent flow is more difficult than pulsing them in a vacuum system.

Finally, the removal of reaction byproducts is passive in S-ALD systems like atmospheric pressure S-ALD (AP-SALD), relying solely on gas flow. In contrast, vacuum purges in temporal ALD actively remove them. As a result, sticky byproducts might not be fully eliminated, potentially contaminating subsequent cycles. These challenges are fundamental to the spatial ALD approach and are shared by its variants, including AP-SALD.

The application of ALD in flexible and emerging devices also presents notable challenges. While the low-temperature nature of ALD is compatible with polymer films, metal foils, and other flexible substrates [92], achieving dense, stable, and defect-free films while maintaining mechanical flexibility and low weight remains a significant hurdle. This challenge directly impacts the broader adoption of ALD in wearable photovoltaics and building-integrated PV systems.

Moreover, intelligent and predictive process optimization is still in its infancy. Although computational fluid dynamics (CFD), density functional theory (DFT), and Gaussian process regression have been applied to optimize reactor design and deposition parameters [210,211], the integration of these methods into a comprehensive framework that correlates precursor flow, temperature, pressure, and other variables with film quality remains underdeveloped. Building a closed-loop system that enables real-time monitoring, parameter adjustment, and performance feedback is crucial for enhancing process stability and scalability.

## 6. Conclusions and Outlook

ALD has established itself as a transformative technique for the fabrication of perovskite solar cells. Its unique capabilities—including precise atomic-level control, low-temperature processing, and unparalleled conformality—make it an indispensable tool for engineering high-quality electron and hole transport layers, effective passivation interfaces, and robust encapsulation. By enabling superior interfacial properties, suppressing charge recombination, and enhancing environmental resilience, ALD has been pivotal in advancing both the power conversion efficiency and operational stability of PSCs to unprecedented levels.

Despite these promising advantages, the path to widespread industrial adoption of ALD faces several formidable challenges. The inherently low deposition rate, high capital investment, and difficulties in integrating with flexible substrates present significant economic and technical hurdles. Moreover, achieving dense, electrically uniform films over large-area and complex device architectures remains a critical bottleneck that must be addressed to meet manufacturing standards.

Future research efforts should focus on overcoming these limitations through synergistic engineering and fundamental innovation. The development of S-ALD and AP-ALD is poised to dramatically improve throughput and scalability, making roll-to-roll processing a realistic prospect. The integration of automation, digital manufacturing, and intelligent modeling and simulation tools holds the promise to accelerate the optimization of ALD processes and materials, reducing development time and cost. Furthermore, exploring the role of ALD in perovskite-based tandem architectures and flexible optoelectronics will open new frontiers for next-generation photovoltaic technologies.

In conclusion, ALD is much more than a mere deposition technique; it is a powerful enabling platform for interfacial science and nano-engineering in photovoltaics. As ALD technologies continue to evolve in scalability, affordability, and intelligence, they will not only unlock the full commercial potential of PSCs but also play a vital role in diversifying the photovoltaic landscape and accelerating the global transition to sustainable energy.

## Figures and Tables

**Table 2:** Temperature Limits for thermally unstable layers in perovskite solar cells.

Device Layers	Temperature Range (°C)	Influencing Factors	Refs.
PET	≤150	The glass transition temperature of PET is circa 70 °C. Excessive temperature can cause the substrate to soften or shrink.	[176,177,178]
PI	≤400	PI has high thermal stability but high cost.	[179,180]
PEN	≤150	The glass transition temperature of PET is circa 120 °C.	[176,181]
Perovskite layers	≤80–250	The perovskite layer is highly sensitive to heat and is prone to decomposition or ion migration upon heating. The thermal decomposition temperature of perovskites can vary greatly depending on the preparation method, as well as the composition and ratio of their organic or inorganic components. Moreover, the duration of exposure at the given temperature also has a pronounced effect on their stability.	[72,92,182,183,184,185]

## Data Availability

No new data were created or analyzed in this study.
